# Myeloid-derived growth factor promotes M2 macrophage polarization and attenuates Sjögren’s syndrome via suppression of the CX3CL1/CX3CR1 axis

**DOI:** 10.3389/fimmu.2024.1465938

**Published:** 2024-10-21

**Authors:** Zi Yang, Mangnan Liu, Zhichao Chang, Conglin Du, Yang Yang, Chen Zhang, Liang Hu

**Affiliations:** ^1^ Department of Endodontics, School of Stomatology, Capital Medical University, Beijing, China; ^2^ Salivary Gland Disease Center and Beijing Key Laboratory of Tooth Regeneration and Function Reconstruction, School of Stomatology and Beijing Laboratory of Oral Health, Beijing, China; ^3^ Department of Oral and Maxillofacial & Head and Neck Oncology, School of Stomatology, Capital Medical University, Beijing, China; ^4^ Outpatient Department of Oral and Maxillofacial Surgery, School of Stomatology, Capital Medical University, Beijing, China

**Keywords:** Sjögren’s syndrome, salivary glands, myeloid-derived growth factor, M2 macrophage polarization, CX3CL1, CX3CR1

## Abstract

**Introduction:**

Primary Sjögren syndrome (pSS) is a systemic autoimmune disease that is characterized by the infiltration of immune cells into the salivary glands. The re-establishment of salivary glands (SGs) function in pSS remains a clinical challenge. Myeloid-derived growth factor (MYDGF) has anti-inflammatory, immunomodulatory, and tissue-functional restorative abilities. However, its potential to restore SGs function during pSS has not yet been investigated.

**Methods:**

Nonobese diabetic (NOD)/LtJ mice (pSS model) were intravenously administered with adeno-associated viruses carrying MYDGF at 11 weeks of age. Salivary flow rates were determined before and after treatment. Mice were killed 5 weeks after MYDGF treatment, and submandibular glands were collected for analyses of histological disease scores, inflammatory cell infiltration, PCR determination of genes, and Western blotting of functional proteins. Furthermore, mRNA sequencing and bioinformatics were used to predict the mechanism underlying the therapeutic effect of MYDGF.

**Results:**

Treatment of NOD/LtJ mice with MYDGF alleviated pSS, as indicated by increased salivary flow rate, reduced lymphocyte infiltration, attenuated glandular inflammation, and enhanced AQP5 and NKCC1 expression. The gene expression levels of cytokines and chemokines, including *Ccl12, Ccl3, Il1r1*, *Ccr2*, *Cx3cr1*, *Il7*, *Mmp2*, *Mmp14*, *Il1b*, and *Il7*, significantly decreased after treatment with MYDGF, as determined by RNA sequencing. Meanwhile, MYDGF inhibits infiltration of macrophages (Mϕ) in SGs, induces polarization of M2ϕ, and suppresses C-X3C motif ligand 1 (CX3CL1)/C-X3C motif receptor 1 (CX3CR1) axis.

**Conclusions:**

Our findings showed that MYDGF could revitalize the SGs function of pSS, inhibit infiltration of Mϕ, and promote M2ϕ polarization via suppression of the CX3CL1/CX3CR1 axis, which has implications for potential therapy for pSS.

## 1 Introduction

Primary Sjögren syndrome (pSS) is a systemic autoimmune disease that affects the salivary and lacrimal glands, resulting in xerostomia and xerophthalmia. The prognosis of the disease leads to systemic complications in approximately 20%–50% of patients with pSS (1). Immunosuppressive and anti-inflammatory therapy (2), regenerative therapy (3), and gene therapy (4) have been reported to alleviate xerostomia of pSS. There is no gold standard for xerostomia treatment currently, and effective clinical treatment for pSS is still in high demand.

The salivary glands (SGs) of pSS patients are characterized by infiltration of immune cells, especially T cells, B cells, dendritic cells (DCs), macrophages (Mϕ), and natural killer (NK) cells (5). Patients with pSS showed increased levels of several Mϕ- and lymphocyte-derived cytokines, such as IL-1b, IL-6, IL-17, MMP3, and TNF-α, indicating an immune activation state (5, 6). Several chemokines, such as CC motif ligand 2 (CCL2)/CC motif receptor 2 (CCR2) and C-X3C motif ligand 1 (CX3CL1)/C-X3C motif receptor 1 (CX3CR1) are associated with lymphocyte homing and vary from one tissue site to another (7, 8). These chemokines are reported to associate with the severity of pSS.

Bone marrow-derived cells (BMDCs), a pool of progenitor and pluripotent stem cells, secrete various cytokines, growth factors, and exosomes. Recent studies have shown that BMDCs can re-establish functions in pSS (9, 10). Given the tissue-specific responses of BMDCs, an increasing number of studies have focused on their paracrine effects as alternative cell-free treatments for various diseases (11, 12). Myeloid-derived growth factor (MYDGF) is a paracrine protein produced by BMDCs, particularly by bone marrow-derived monocytes and Mϕ. Our previous study showed that MYDGF relieves inflammation of inflammatory bowel disease (13). Moreover, MYDGF inhibits inflammation, blunts leukocyte homing, and protects endothelial injury via nuclear factor κB (NF-κB) signaling (14, 15). However, the effects of MYDGF on pSS and its possible underlying mechanisms remain unknown.

In the present study, we used nonobese diabetic (NOD)/LtJ female mice as an animal model of pSS. The function of SGs following MYDGF treatment was analyzed, and the underlying molecular mechanisms of MYDGF were investigated. The present study reveals the function and underlying mechanism of interactions between MYDGF and the host immune system in pSS, providing potential strategies for improving the therapeutic efficacy of pSS.

## 2 Materials and methods

### 2.1 Animal experiments

Female Institute of Cancer Research (ICR) and NOD/LtJ 6-week-old mice purchased from Beijing Vital River Laboratory Animal Technology (Beijing, China) were used as normal and the pSS model subjects. All mouse experiments were approved by the Ethics Review Commission of the Laboratory Animal Center of Capital Medical University (approval number: AEEI-2023-056). The mice had free access to soy-based food and water and were kept under a light/dark cycle for 12/12 h at a constant temperature.

### 2.2 Adeno-associated-virus -treated mice

The *Mydgf* gene sequence (GenBank accession number NM 080837.2) was directly synthesized into the CMV-betaGlobin-EGFP-T2A-MCS-3Flag-SV40 PolyA vector. The mice received a single injection of adeno-associated virus (AAV)-MYDGF or AAV-GFP at a dose of 1 × 10^12^ viral genomes through the tail vein for 11 weeks. The ICR mice were designated as the normal group and did not receive any treatment. NOD/LtJ mice were categorized into the following groups based on the material used for intravenous injection (*n* = 8 per group): (1) SS group, not administered anything; (2) AAV-GFP group, AAV-GFP administered; and (3) AAV-MYDGF group, AAV-MYDGF administered. Saliva secretion flow rates for all four groups were recorded at weeks 8, 11, 14, and 16. Mice were killed at week 16, and submandibular glands, spleen, and blood were collected for further experiments.

### 2.3 Measurement of stimulated saliva flow

The ICR and NOD/LtJ mice were anesthetized with pentobarbital sodium (50 mg/kg). After an intraperitoneal injection of pilocarpine (50 mg/mL) at a dose of 0.1 mL/kg body weight, stimulated saliva flow was measured 10 min later. A cotton ball was placed under the tongue and held steadily during a 10-min period to collect saliva. The weight difference of the cotton ball before and after saliva collection was calculated.

### 2.4 Hematoxylin and eosin staining

At 16 weeks, all animals were killed, and the submandibular glands were harvested. Portions of the submandibular glands were fixed in 4% paraformaldehyde and then embedded in paraffin. Four-micrometer sections were obtained, deparaffinized, hydrated, and stained with hematoxylin and eosin (HE) and for immunofluorescence. The counts and areas of inflammatory foci containing > 50 lymphocytes per 4 mm^2^ of tissue were calculated.

### 2.5 Immunofluorescence and immunohistochemistry (IHC) staining

The sections were dewaxed and rehydrated for immunohistochemistry with xylol and alcohol, respectively. Antigen retrieval was conducted by sodium citrate in microwave conditions. The slides were further blocked with 3% bovine serum albumin (BSA; Beyotime, Shanghai, China) for 1 h at 37°C. Primary antibodies, including anti-IL-6 (1:100, ab290735, Abcam, Waltham, MA, USA), anti-TNF-α (1:100, ab6671, Abcam), anti-AQP5 (1:1,000, ab305303, Abcam), anti-NKCC1 (1:200, 13884-1-AP, Proteintech, Wuhan, China), anti-F4/80 (GB113373, Servicebio, Wuhan, China), anti-CD86 (GB115630, Servicebio), anti-CD206 (GB113497, Servicebio), anti-CX3CL1 (60339-1-1g, Proteintech), anti-CX3CR1 (13885-1-AP, Proteintech), were incubated with the slides at 4°C overnight. Secondary antibodies (1: 1,000, A32723, A-11012, Thermo Fisher, Waltham, MA, USA) and Fluoroshield™ with 4',6-diamidino-2-phenylindole (DAPI) (Sigma-Aldrich Corp., St. Louis, MO, USA) were used for immunofluorescence (IF) staining. The secondary antibody (PV-9000, ZSGB-Bio, Beijing, China) and DAB kit (8059P, Cell Signaling Technology, Boston, USA) were used in immunohistochemistry (IHC) staining. Stained samples were evaluated using a fluorescence microscope (BX61, Olympus, Tokyo, Japan).

### 2.6 Western blot

Portions of the submandibular glands were ground and prepared with lysis buffer (C1053, Applygen, Beijing, China) according to the manufacturer’s instructions. Protein concentrations were determined by Coomassie Brilliant Blue (1610435, Bio-Rad, Hercules, CA, USA). Equal amounts of cellular proteins (30 μg) were boiled for 10 min at 98°C. Equal amounts of protein extracts were loaded onto FuturePAGE™ 4%–20% polyacrylamide gels (ACE, Nanjing, China) for electrophoresis and transferred to nitrocellulose membranes. Bands were detected immunologically using polyclonal antibodies (1: 1,000) against AQP5 (ab305303, Abcam), MYDGF (11353-1-AP, Proteintech), and β- actin (AC026, Abclonal, Wuhan, China), which were used as a loading control. Immunoblot bands were visualized following the application of the ECL detection system (ChemiDoc™ MP Imaging System, Bio-Rad, CA, USA).

### 2.7 Flow cytometry

The spleens of the AAV-GFP and AAV-MYDGF group were ground and filtrated through a Falcon 70 μm filter (Corning Inc., Corning, NY, USA). R ed blood cell lysis buffer (R1010, Solarbio, Beijing, China) was then added, and the mixture was centrifuged at 500×*g*. A single-cell suspension was stained with the following antibodies: CD4 (100559, BV510, RM4-5, Biolegend San Diego, CA, USA), CD45 (147716, Alexa Fluor 700, I3/2.3, Biolegend), TCRβ (109220, APC-Cyanine7, H57-597, Biolegend), F4/80 (123131, BV421, BM8, Biolegend), CD80 (104705, FITC, 16-10A1, Biolegend), CD163 (155305, APC, S150491, Biolegend), IL-4 (504103, PE, 11B11, Biolegend), IL-17A (506922, PE-Cyanine7, TC11-18H10.1, Biolegend), FOXP3 (126409, Pacific Blue, MF-14, Biolegend), and IFN-γ (505817, Pacific Blue, XMG1.2, Biolegend). For intracellular cytokine staining, cells were stimulated with a cell stimulation cocktail (00-4970-93, Invitrogen, Waltham, MA, USA) and a protein transport inhibitor cocktail (00-4980-03, Invitrogen) at 37°C for 6 h, followed by fixation with the fixation/permeabilization buffer solution (554714, BD Bioscience, New jersey, USA). Foxp3/Transcription Factor Fixation/Permeabilization Concentrate and Diluent (00-5521, Invitrogen) were used for intranuclear staining. Stained cells were analyzed using an LSRFortessa (BD Biosciences), and the data were analyzed using FlowJo software (Tree Star, Ashland, OR, USA).

### 2.8 RNA sequencing

Samples of submandibular glands from the AAV-GFP and AAV-MYDGF groups were collected for RNA sequencing. The preparation of transcriptome libraries and sequencing were performed by OE Biotech Co. Ltd. (Shanghai, China). A comparison was conducted to identify genes that were differentially regulated between the AAV-GFP and AAV-MYDGF groups. A *p*-value < 0.05 and a fold change > 1.5 or < 0.75 were set as the thresholds for significant differential expression. The Gene Ontology (GO) enrichment analysis, Kyoto Encyclopedia of Genes and Genomes (KEGG) enrichment analysis, and Gene Set Enrichment Analysis (GSEA) were performed using R based on a hypergeometric distribution.

### 2.9 Quantitative real-time polymerase chain reaction

Portions of the submandibular glands were ground, and total RNA was collected using the RNAprep pure Tissue Kit (DP431, Tiangen Biotech, Beijing, China). The RNA was then reverse-transcribed into cDNA using the NovoScript^®^ Plus All-in-one 1st Strand cDNA Synthesis SuperMix (E047-01A; Novoprotein, Suzhou, China). Quantitative real-time polymerase chain reaction (qRT-PCR) was performed using an NovoStart^®^SYBR qPCR SuperMix Plus (E096-01B; Novoprotein). After normalizing target gene expression, the data were quantified using the 2^−ΔΔCt^ method. The genes and their corresponding primer sequences are listed in **Supplementary Table S1**.

### 2.10 Enzyme-linked immunosorbent assay

Blood samples were collected after the animals were anesthetized. Serum was obtained by centrifugation at 1,500 rpm for 30 min at room temperature and then stored at − 20°C. The concentrations of CX3CL1 and CX3CR1 in serum were detected by mouse CX3CL1 and CX3CR1 enzyme-linked immunosorbent assay (ELISA) Kit (SYP-M0090, SYP- M0768, UpingBio Technology, Shenzhen, China).

### 2.11 Terminal deoxynucleotidyl transferase dUTP nick end labeling assay

Terminal deoxynucleotidyl transferase dUTP nick end labeling (TUNEL) staining was performed using a One- step TUNEL Apoptosis Assay Kit (C1090; Beyotime, Shanghai, China). Images were captured under a confocal microscope, and the number of TUNEL-positive cells was calculated using Image J software (NIH, Bethesda, MD, U SA).

### 2.12 Statistical analysis

All results are presented as mean ± SDs. The one-way ANOVA test was used to determine significant differences among multiple groups, while the Kruskal–Wallis test was used to compare differences with non-normal distribution. Student’s *t*-test was performed to compare differences between two groups with normal distribution. Statistical analysis was performed using SPSS 19.0 and by GraphPad Prism 8 software (San Diego, CA, USA). In the figures, asterisks denote statistical significance as follows: ^ns^ *p* > 0.05, ^*^ *p* < 0.05, ^**^ *p* < 0.01, and ^***^ *p* < 0.001.

## 3 Results

### 3.1 MYDGF treatment alleviates xerostomia and revitalizes SG function in NOD/LtJ mice

**Figure 1:**
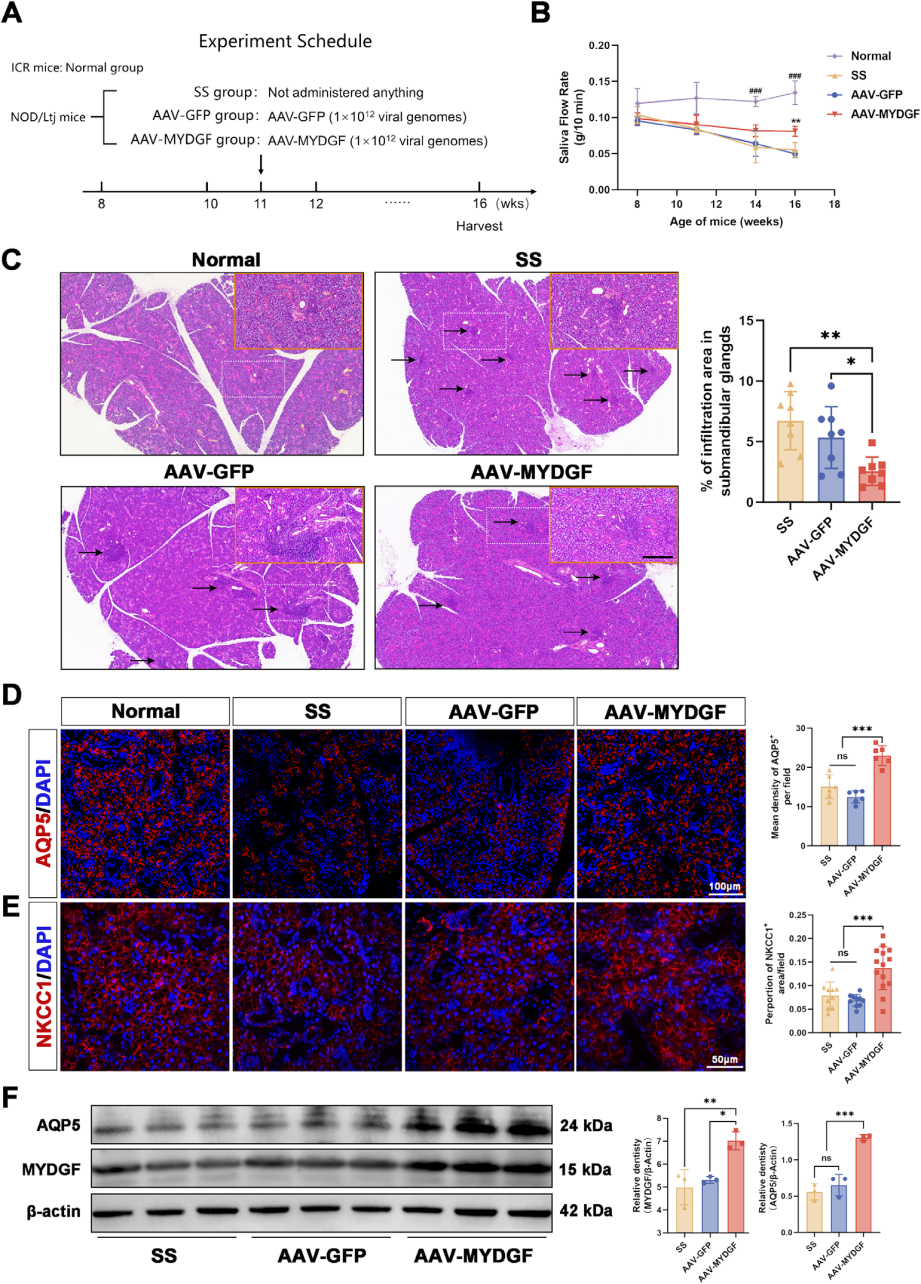
MYDGF alleviated the function of salivary glands in NOD/Ltj mice. **(A)** Schematic of the animal experiment and treatment. **(B)** Saliva flow rate (g/10 min) of four groups (^**^ *p* < 0.01 vs. AAV-GFP and SS group; ^###^ *p* < 0.001 vs. SS, AAV- GFP, and AAV-MYDFG groups). **(C)** HE-stained histological images of submandibular glands and quantitative analysis of infiltration area showed that the number and area of lymphocyte infiltration foci (black arrows) were considerably reduced in the AAV-MYDGF group than those in the SS and AAV-GFP groups (scale bars: 500 μm and 200 μm; ^ns^ no significant difference; ^***^ *p* < 0.001). **(D)** Immunofluorescence staining of AQP5 (red) and quantitative analysis showed the expression of AQP5 was strongly increased in the AAV-MYDGF group compared with the SS and AAV-GFP groups (scale bar: 100 μm, *** *p* < 0.001). **(E)** Immunofluorescence staining of NKCC1 (red) and quantitative analysis showed the expression of NKCC1 was significantly increased in the AAV-MYDGF group (scale bar: 50 μm, ^***^ *p* < 0.001). **(F)** Western blot analysis showed that the expression of AQP5 and MYDGF in theAAV-MYDGF group was significantly increased compared with the SS and AAV-GFP groups (^*^ *p* < 0.05; ^**^ *p* < 0.01; ^***^ *p* < 0.001). One-way analysis of variance (ANOVA) was used to compare the differences among multiple groups, with the Bonferroni method applied to used to compare the intergroup variation.

NOD/LtJ mice were used as a spontaneous pSS model, exhibiting characteristic lymphocyte infiltration in the exocrine glands and SG dysfunction. The animal experiments were conducted as illustrated in **Figure 1A**. A decrease in salivary secretion was observed between the fifth and eighth weeks, after which it was maintained at a low level (16). At the 11th week, the salivary flow rate in NOD/LtJ mice significantly decreased compared to that in ICR mice (**Figure 1B**, *p* < 0.05), indicating that the early stage of the pSS model had been established. To assess the effect of MYDGF during the early stages of pSS, AAV-GFP and AAV-MYDGF were injected in the 11th week. After 5 weeks of AAV-MYDGF treatment, salivary function and histologic examination were performed in the 16th week.

The saliva flow rate in the SS and AAV-GFP groups was reduced at 16 weeks compared to that at 8 weeks. However, MYDGF treatment significantly maintained the saliva flow rate compared to that of the SS and AAV-GFP groups (**Figure 1B**, *p* < 0.01). As indicated by HE staining, the number and area of lymphocyte infiltration foci in the submandibular glands were significantly reduced in the AAV-MYDGF group compared to those in the SS and AAV-GFP groups (**Figure 1C**, *p* < 0.01). Aquaporin-5 (AQP5) and the Na^+^/K^+^/2Cl ^−^ cotransporter (NKCC1) are two important transmembrane transporter proteins involved in salivary secretion in SG acinar cells. Both proteins were significantly reduced at the onset of SS, which correlated with decreased salivary secretion (17–19). Immunofluorescence (IF) staining showed that AQP5 and NKCC1 were widely expressed in the acinar cells of the submandibular glands of ICR mice, and their expression in the membrane of acinar cells was strongly enhanced in the AAV-MYDGF group compared to that in the SS and AAV-GFP groups (**Figures 1D, E**, *p* < 0.001). Western blotting showed similar protein expression levels of AQP5 and MYDGF in the AAV-MYDGF group, supporting our results (**Figure 1F**, *p* < 0.05 or *p* < 0.01). Overall, MYDGF treatment inhibited lymphocyte infiltration and restored salivary secretion during the early stages of pSS.

### 3.2 MYDGF ameliorates inflammation and chemokines of SGs in NOD/LtJ mice

**Figure 2:**
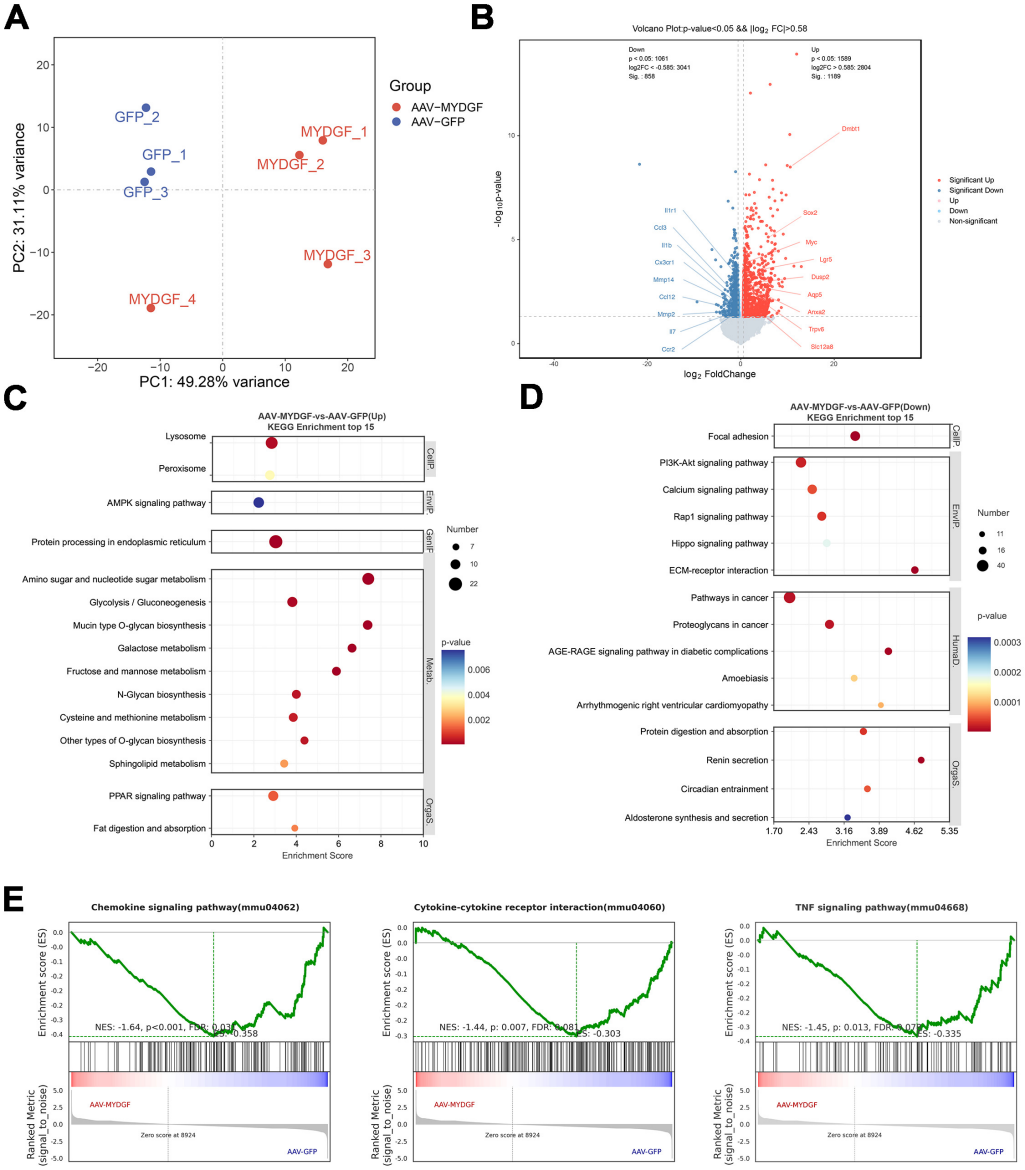
Transcriptome analysis of submandibular glands of NOD/Ltj mice treated with AAV-GFP and AAV-MYDGF. **(A)** The principal component analysis (PCA) showed the distinct separation between the AAV-GFP and AAV-MYDGF group. **(B)** Volcano plot of differentially expressed genes (DEGs) showed that the gene expression of chemokines, such as *Ccl12*, *Ccl3*, *Il1r1*, *Ccr2*, and *Cx3cr1* were significantly decreased in the AAV-MYDGF group (*p* < 0.05). **(C, D)** Top 15 KEGG enrichment. **(E)** GSEA showed that TNF signaling pathway, cytokine-cytokine receptor interaction and chemokine signaling pathway were significantly downregulated in the AAV-MYDGF group.

Submandibular gland samples from the AAV-GFP and AAV-MYDGF groups were subjected to RNA sequencing to investigate the underlying mechanisms of MYDGF treatment in pSS. Principal component analysis (PCA) showed a distinct separation between the two groups, confirming the comparability of the samples (**Figure 2A**). Furthermore, a total of 2,047 differentially expressed genes (DGEs) were identified in the AAV-MYDGF group compared to the AAV-GFP group, with 1,189 upregulated and 858 downregulated. Notably, the expression levels of chemokines associated with the severity of pSS, such as *Ccl12*, *Ccl3*, *Il1r1*, *Ccr2*, *Cx3cr1*, *Il7*, *Mmp2*, *Mmp14*, *Il1b*, and *Il7*, were significantly lower in the AAV- MYDGF group than in the AAV-GFP group. Genes associated with the function of SGs, including *Aqp5*, *Sox2*, *Slc12a8*, *Lgr5*, and *Dusp2*, were significantly upregulated in the AAV-MYDGF group (**Figure 2B**, *p* < 0.05).

**Figure 3:**
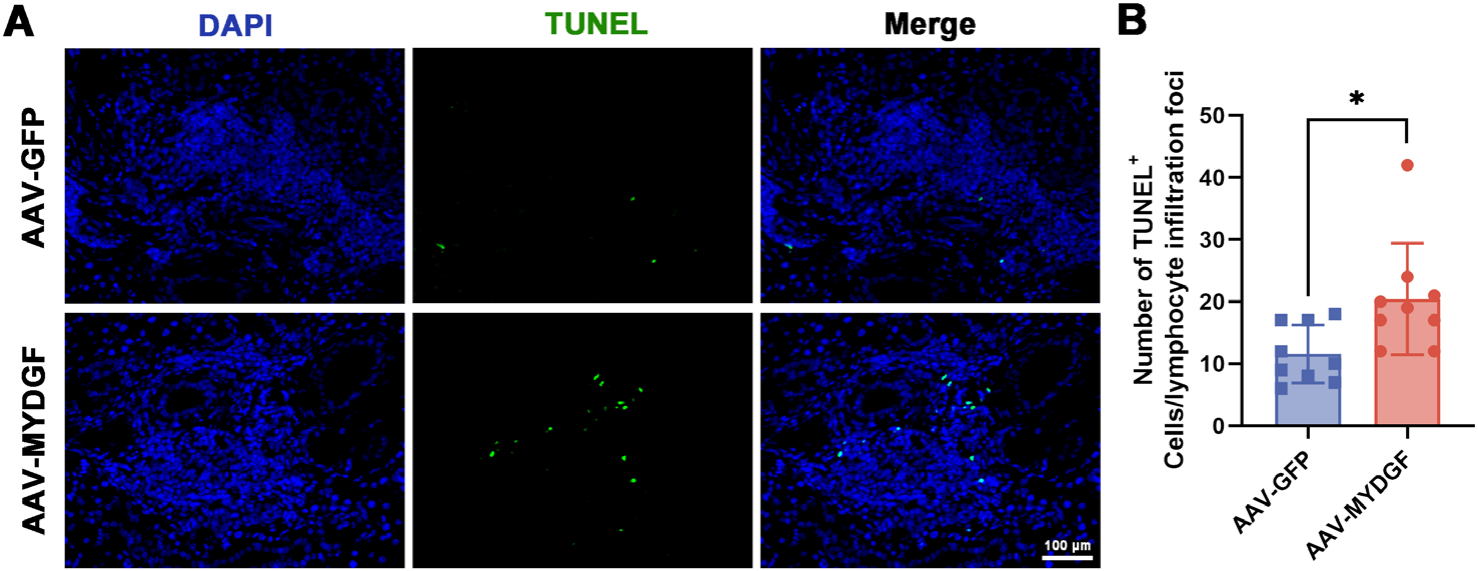
MYDGF promotes the apoptosis of immune cells of lymphocyte infiltration foci. **(A, B)** The TUNEL staining showed that the number of TUNEL^+^ cells in lymphocyte infiltration foci of submandibular glands was significantly increased in the AAV-MYDGF group (scale bar: 100 μm, ^*^ *p* < 0.05). Student’s *t*-test was performed to compare difference between two groups with normal distribution.

GO analysis revealed that MYDGF treatment upregulated biological processes, including neutral amino acid transport, vesicle-mediated transport, protein transport, and endoplasmic reticulum-to-Golgi vesicle-mediated transport (**Supplementary Figures S1A, B**). Additionally, KEGG analysis showed that metabolism-related signaling pathways were upregulated, while the PI3K-AKT and Hippo signaling pathways were downregulated following MYDGF treatment (**Figures 2C, D**, *p* < 0.05). GSEA showed that TNF signaling pathway, cytokine-cytokine receptor interaction and chemokine signaling pathway were significantly downregulated in the AAV-MYDGF group (**Figure 2E**, *p* < 0.05). In addition, IHC staining showed that the expression levels of TNF- α and IL-6 were significantly decreased in the AAV-MYDGF group (**Supplementary Figures S2A–D**; *p* < 0.001), indicating that MYDGF inhibits inflammation and revitalizes SG function by modulating the secretion of cytokines and chemokines. TUNEL staining revealed a significant increase in the number of TUNEL^+^ cells within the lymphocyte infiltration foci in the salivary glands of the AAV-MYDGF group compared to the AAV-GFP group (**Figures 3A, B**, *p* < 0.05). Overall, MYDGF alleviated inflammation, inhibited the expression of chemokines, and promoted the necrosis of lymphocytes infiltrating the submandibular glands.

### 3.3 MYDGF inhibits infiltration of Mϕ in SGs and induces polarization of M2ϕ

An imbalance in T helper cells contributes to the pathogenesis of pSS by producing proinflammatory cytokines (18, 20, 21). The ratio of T helper lymphocyte subsets in the spleen was determined using flow cytometry. The results showed that the ratios of Th1 (CD4^+^/IFN-γ^+^) or Th2 (CD4^+^/IL4^+^) cells, representing Th1 or Th2 cells, respectively, were not significantly different between the AAV-MYDGF and AAV-GFP groups (**Supplementary Figures S3A, B**, *p* > 0.05). Additionally, the ratio of Th17 (CD4^+^/RORg^+^) to Treg (CD4^+^/CD25^+^/FOXP3^+^) cells was not significantly different between the AAV-MYDGF and AAV-GFP groups (**Supplementary Figures S3C, D**, *p* > 0.05).

**Figure 4:**
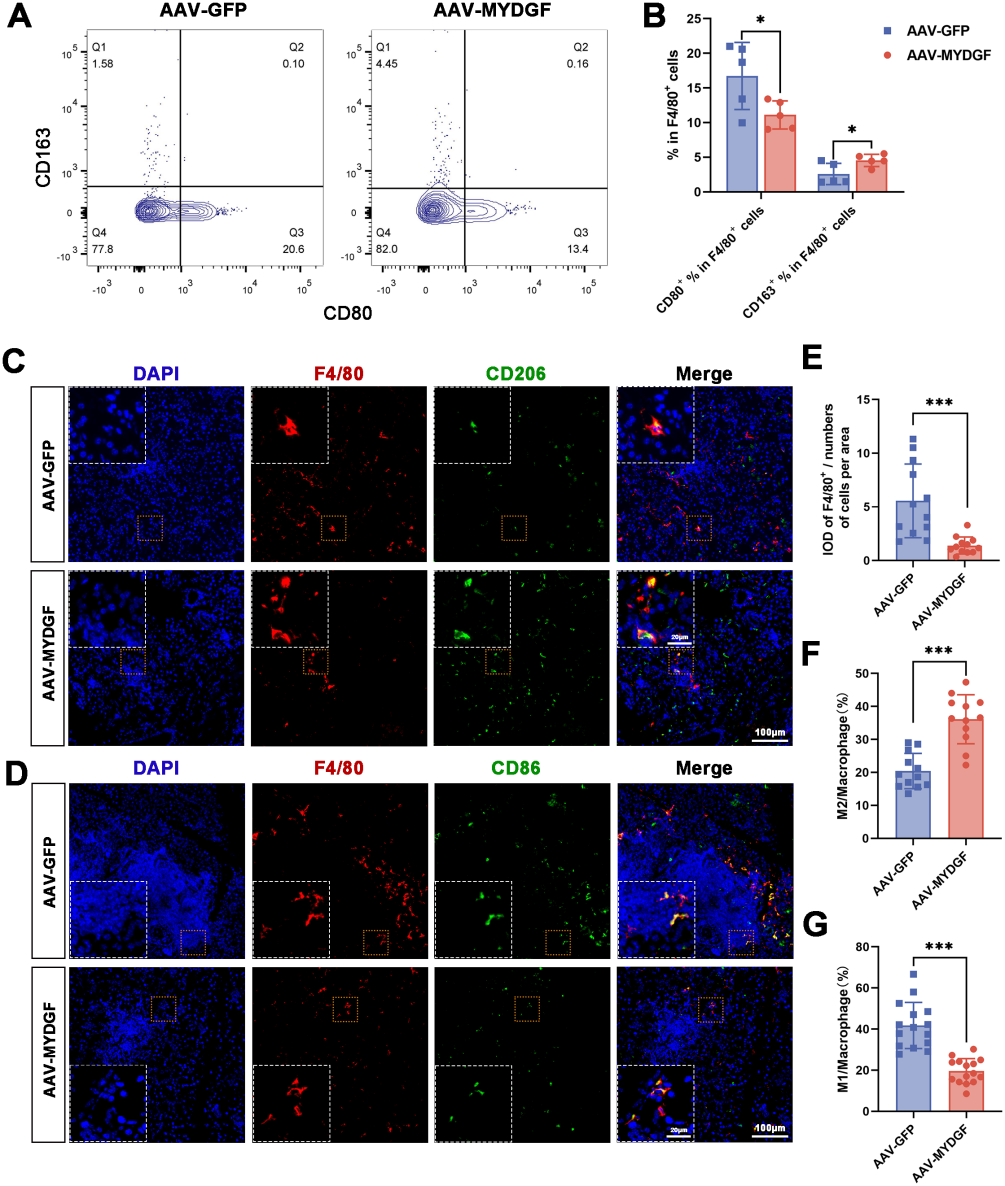
MYDGF inhibits the infiltration of Mϕ in the submandibular glands and induces polarization of M2ϕ. **(A, B)** Flow cytometry of the spleen showed that the ratio of M2ϕ (F4/80^+^/CD163^+^) was increased and the ratio of M1ϕ (F4/80^+^/CD80^+^) was decreased after treatment of MYDGF (^*^ *p* < 0.05). **(C, E, F)** Double immunofluorescence of the submandibular glands with DAPI (blue), F4/80 (red), and CD206 (green) revealed that the numbers of positive of F4/80^+^ cells was significantly decreased, and the ration of M2ϕ was significantly increased in the AAV-MYDGF group compared with the AAV-GPF group (scale bar: 100 and 20 μm, ^***^ *p* < 0.001). **(D, G)** Double immunofluorescence of the submandibular glands with DAPI (blue), F4/80 (red), and CD86 (green) revealed that the ratio of M1ϕ was significantly decreased in the AAV-MYDGF group compared with the AAV-GPF group (scale bar: 100 and 20 μm, ^***^ *p* < 0.001). Student’s *t*-test was performed to compare difference between two groups with normal distribution.

Mϕ, a crucial component of the innate immune system, correlates with pSS severity (22). Flow cytometry analysis of the spleen revealed that treatment with MYDGF increased the ratio of M2ϕ (F4/80^+^/CD163^+^) and decreased the ratio of M1ϕ (F4/80^+^/CD80^+^) (**Figures 4A, B**, *p* < 0.05). Based on immunohistochemical staining of submandibular glands, the number of positive F4/80^+^ cells was significantly decreased in the AAV-MYDGF group compared to the AAV-GPF group (**Figures 4C–E**, *p* < 0.001). Double immunofluorescence staining of F4/80 and CD206 indicated that MYDGF treatment significantly promoted the polarization of M2ϕ compared to the AAV-GPF group (**Figures 4C, F**, *p* < 0.001). Meanwhile, IF of F4/80 and CD86 showed that MYDGF treatment significantly decreased the polarization of M1ϕ (**Figures 4D, G**, *p* < 0.001). In summary, MYDGF suppresses the infiltration of Mϕ and promotes M2ϕ polarization in pSS.

### 3.4 MYDGF inhibits inflammation via suppression of the CX3CL1/CX3CR1 axis

**Figure 5:**
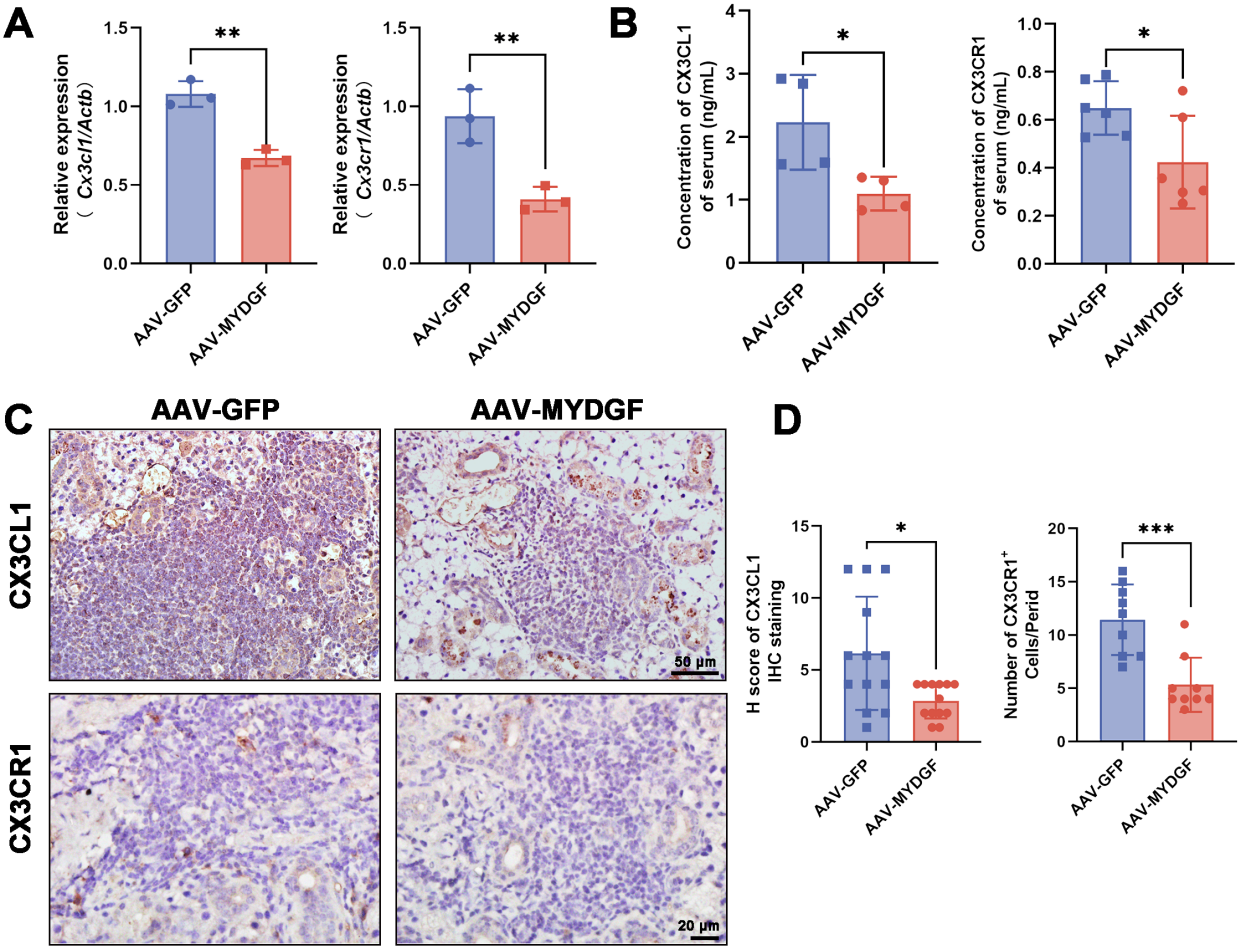
MYDGF inhibits inflammation via suppression of the CX3CL1/CX3CR1 axis. **(A)** RT-PCR showed that the mRNA expression of *Cx3cl1* and *Cx3cr1* of SGs was significantly decreased after treatment of MYDGF (^**^ *p* < 0.01). **(B)** The concentration of CX3CL1 and CX3CR1 of serum measured by ELISA was significantly decreased after treatment of MYDGF (^*^ *p* < 0.05). **(C, D)** Immunohistochemistry staining and quantitative analysis showed that MYDGF could decrease the expression of CX3CL1 and CX3CR1 of SGs (^*^ *p* < 0.05; ^***^ *p* < 0.001). Student’s *t*-test was performed to compare differences between two groups with a normal distribution. The Kruskal–Wallis test was employed to compare differences with non-normal distribution.

Based on RNA sequencing, we found that the expression of *Cx3cr1* was significantly decreased in the AAV-MYDGF group (**Figure 2B**, *p* < 0.05). RT-PCR showed that after treatment with MYDGF, the expression of chemokine- or cytokine-related genes, including *Ccl2*, *Ccl3*, *Ccl12*, *Mmp2*, *Cx3cl1*, and *Cx3cr1*, was significantly decreased, with *Cx3cl1* and *Cx3cr1* showing the most pronounced reduction (**Figure 5A**; **Supplementary Figure S4**, *p* < 0.01). The serum concentrations of CX3CL1 and CX3CR1 significantly decreased after MYDGF treatment (**Figure 5B**, *p* < 0.05). IHC staining of submandibular glands showed that MYDGF decreased the expression of CX3CL1 and CX3CR1 in SGs (**Figures 5C, D**, *p* < 0.05). CX3CL1/CX3CR1 were the most significantly decreased chemokines in serum and submandibular glands after treatment of MYDGF.

## 3 Discussion

pSS is a chronic autoimmune disease that affects the exocrine glands and causes systemic autoimmune lesions (23). Characteristics of pSS include inflammatory cell infiltration and increased cytokine and chemokine production. However, the pathogenesis of pSS is not fully understood, and effective clinical therapies are still limited. MYDGF, a paracrine protein of BMDCs, inhibits inflammation and blunts immune cell homing and migration (13–15). Further research is required to determine whether MYDGF can be used as an alternative cell-free therapy to alleviate xerostomia in patients with pSS. In the present study, MYDGF demonstrated the ability to inhibit inflammation, reduce the migration of lymphocytes and Mϕ in SGs, and re-establish the impaired SG function of pSS. In addition, we found that MYDGF promote d the polarization of M2ϕ and suppressed the expression of CX3CL1 and CX3CR1 in both the spleen and SGs. Our results indicate the therapeutic potential and molecular mechanisms of MYDGF in pSS.

Previous studies have shown that pSS is triggered by a T-cell-mediated autoimmune response (24) and B-cell activation (25); however, other immune cells, including Mϕ, have also been observed in SGs and peripheral blood mononuclear cells (PBMCs), contributing to the onset or development of SS (26). However, the role of Mϕ in SS has not been widely investigated. Mϕ has been found to generate reactive oxygen species and communicate with other innate and adaptive immune cells in SGs (27). CD11b^Hi^ Mϕ promotes CCR4 expression in CD4^+^ T cells, thereby improving the migratory capacity in pSS (23). Additionally, the transplantation of CD4^+^ T cells also induces Mϕ infiltration, and the depletion of Mϕ is sufficient to ameliorate the dysfunction of the lacrimal glands and eyes (28). Therefore, targeting Mϕ infiltration is a promising treatment strategy for pSS.

Meanwhile, the polarization of M1ϕ may play a significant role in the development of pSS and could be associated with disease activity. The expression of CD86^+^ M1ϕ is significantly higher, while that of CD206^+^ M2ϕ is significantly lower in the SGs and PBMCs of pSS patients compared to non-pSS controls (22). In the PBMCs of pSS patients, the high abundance of proinflammatory M1ϕ is accompanied by an increased presence of other immune cells, including T cells, B cells, and DCs (26). The portions of CXCR3^+^CD163^+^ M2ϕ decreased as disease severity increased, indicating that M2ϕ may contribute to the progression of pSS (29). Therefore, modulating the imbalance of Mϕ polarization could potentially serve as a promising therapeutic method to ameliorate pSS.

BMDCs release a broad range of soluble factors in a paracrine manner that may promote tissue protection and repair (30). MYDGF, also known as C19orf10, is abundantly found in various cellular microenvironments, including the calcium-rich endoplasmic reticulum and Golgi apparatus (31). It can be secreted and released from BMDCs as a potential paracrine active factor in response to stress (32). Recent studies have shown that MYDGF has potent cardiac myocyte-protective and angiogenic activities, offering protection against cardiovascular and metabolic diseases (14, 32–34). MYDGF reduces inflammation (TNF-a, IL-1β, and IL-6) and leukocyte homing, as well as Mϕ accumulation within aortic plaques (14). The present study confirmed that MYDGF alleviated the inflammatory response (IL-6 and TNF-α) in SGs, downregulated the TNF signaling pathway, and promoted immune cell apoptosis of the lymphocyte infiltration foci, thereby re-establishing the function of SGs. Our results demonstrated that MYDGF significantly reduces the infiltration of Mϕ. Consistent with this finding, MYDGF has a direct effect on Mϕ, as it has been reported to reduce their inflammation and migration (14). In addition, MYDGF enhance s the numbers and proportions of M2ϕ in a dextran sodium sulfate (DSS)−induced colitis model (13), and it decreases M1ϕ polarization while increasing M2ϕ polarization in primary Kupffer cells and in nonalcoholic fatty liver disease mouse model (15). Consistent with previous studies, our results demonstrate that MYDGF promote s the polarization of M2ϕ in SGs.

Furthermore, several cytokines and chemokines secreted by Mϕ contribute to the development of pSS (7). CX3CL1, the only member of the CX3C chemokine family, and its sole receptor, CX3CR1, are involved in the recruitment of monocytes/Mϕ and lymphocytes, playing crucial roles in various autoimmune diseases (35). The expression of fractalkine, the soluble chemokine CX3CL1 cleaved from membrane-bound CX3CL1, CX3CR1-expressed T cells, and Mϕ, was significantly elevated in the lacrimal glands of NOD/LtJ mice (36). CX3CL1 expression levels are higher in the serum of patients with SS compared to healthy controls, and CX3CR1^+^ cells are found in close proximity to the inflammatory foci (8). Therefore, CX3CL1/CX3CR1 may serve as a novel tool for evaluating pSS. The present study found that treatment with MYDGF inhibited the expression of cytokines and chemokines, and the expression of CX3CL1 and CX3CR1 in the serum and SGs was significantly downregulated by MYDGF, indicating that MYDGF may reduce infiltration of the immune system by modulating the production of chemokines such as the CX3CL1/CX3CR1 axis.

The results of the present study imply that MYDGF significantly alleviates pSS by reducing inflammation and re- establishing SG function. MYDGF demonstrated the potential to inhibit Mϕ infiltration, suppress M1ϕ polarization, and promote M2ϕ polarization. In addition, MYDGF inhibited the expression of genes related to chemokines and inflammatory factors in the submandibular glands, particularly by significantly suppressing the CX3CL1/CX3CR1 axis, and promoted the apoptosis of immune cells within lymphatic infiltrates. However, the complex signaling mechanisms by which MYDGF inhibits Mϕ migration and promotes M2ϕ polarization remain unclear, and the crucial role of the CX3CL1/CX3CR1 axis or other chemokines in migration and polarization requires further investigation in future research.

## Data Availability

The raw and processed data from RNA-Seq sequencing in this study have been deposited with the national center for biotechnology infromation (NCBI) under accession number PRJNA1169323 (https://www.ncbi.nlm.nih.gov/bioproject/PRJNA1169323).

## Supplementary material

Supplementary Figure 1 Transcriptome analysis of salivary glands of NOD/Ltj mice treated with AAV-GFP and AAV-MYDGF. **(A, B)** GO analysis.

Supplementary Figure 2 MYDGF ameliorates inflammation of SGs in NOD/Ltj mice. **(A, B)** Immunohistochemistry (IHC) staining and quantitative analysis showed that MYDGF could decrease the expression of TNF-a of SGs, *** *P* < 0.001; **(C, D)** IHC staining and quantitative analysis showed that MYDGF could decrease the expression of IL-6 of submandibular glands, *** *P* < 0.001. Scale bars: 100 and 50 μm. Student’s t-test was performed to compare difference between two groups with normal distribution.

Supplementary Figure 3 The ratio of T helper lymphocyte subsets did not differ in the AAV-MYDGF and AAV-GFP groups. Flow cytometry showed the ratio of **(A)** Th1, **(B)** Th2, **(C)** Th17, **(D)** Treg were no significantly difference in the AAV-MYDGF and AAV-GFP group, ^ns^ no significant difference. Student’s t-test was performed to compare difference between two groups with normal distribution.

Supplementary Figure 4 Chemokine- and cytokine-related gene of AAV-GFP and AAV-MYDGF group by qRT-PCR.
