# Supplementary figures and images for: Myeloid-derived growth factor promotes M2 macrophage polarization and attenuates Sjögren’s syndrome via suppression of the CX3CL1/CX3CR1 axis
Source: Front Immunol. 2024 Oct 21;15:1465938. doi: 10.3389/fimmu.2024.1465938 (PMC11532040; doi:10.3389/fimmu.2024.1465938)

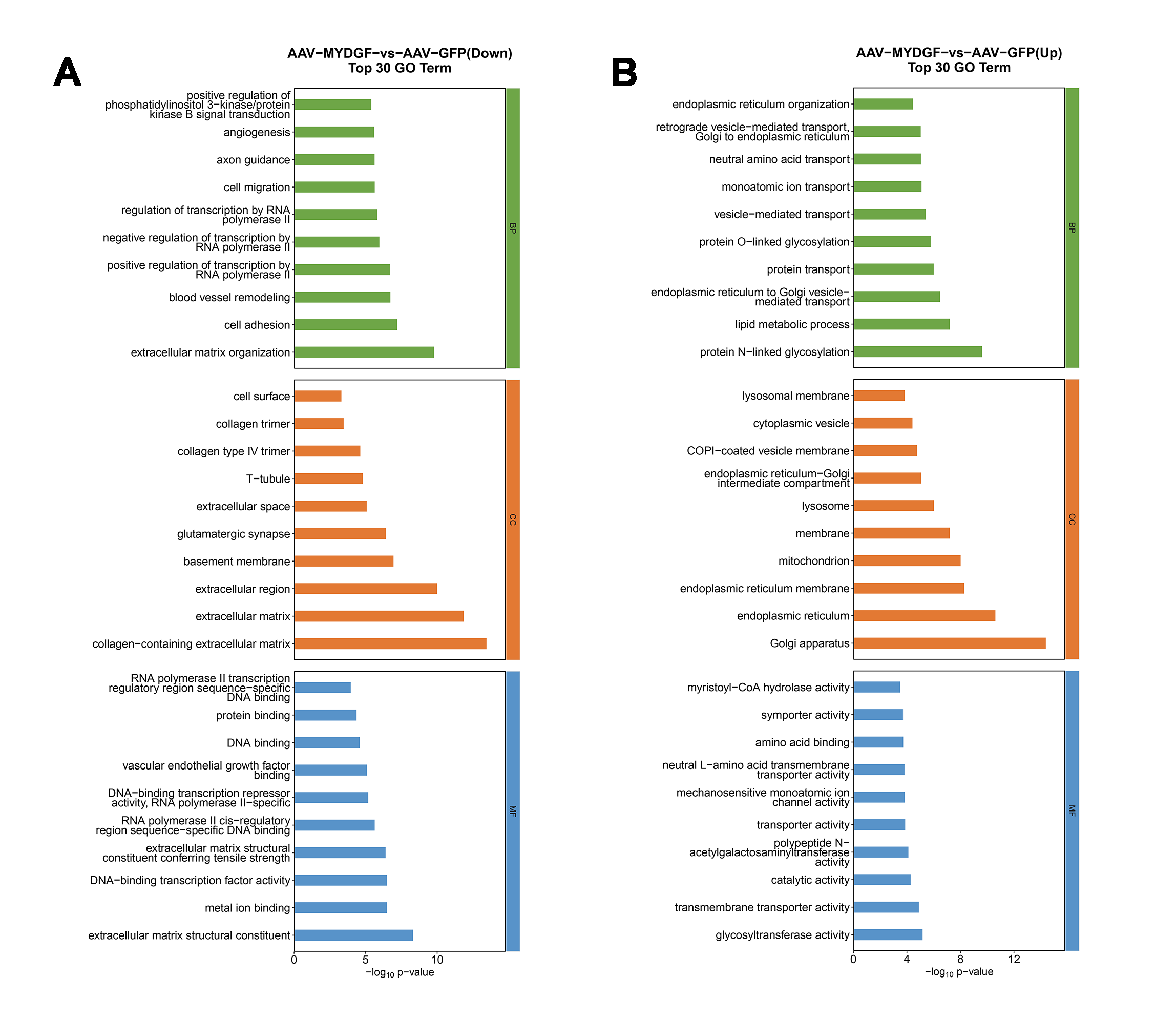

Supplement: Supplementary Figure 1 — Transcriptome analysis of salivary glands of NOD/Ltj mice treated with AAV-GFP and AAV-MYDGF. (A, B) GO analysis. [file Image1.tif]

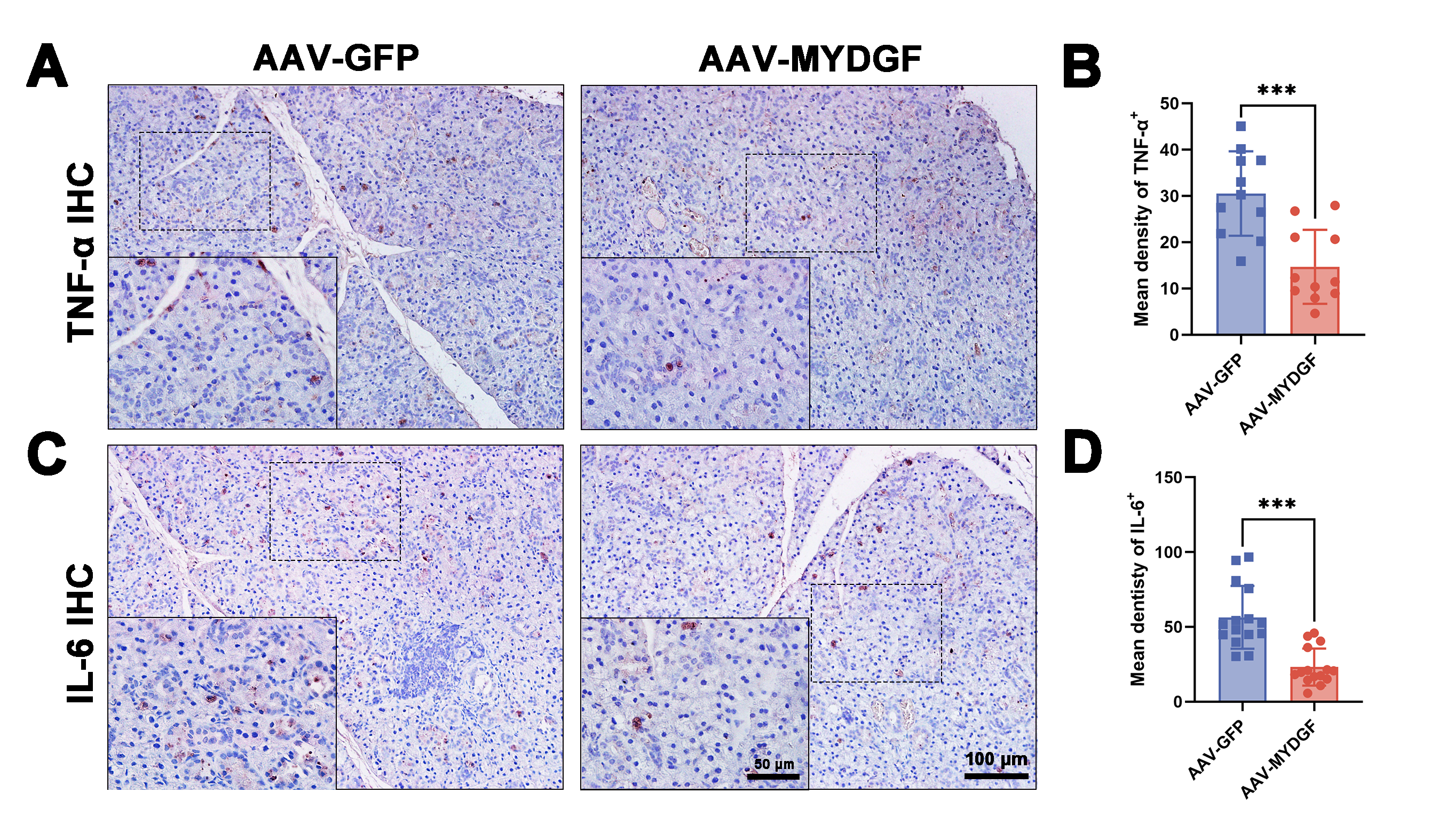

Supplement: Supplementary Figure 2 — MYDGF ameliorates inflammation of SGs in NOD/Ltj mice. (A, B) Immunohistochemistry (IHC) staining and quantitative analysis showed that MYDGF could decrease the expression of TNF-a of SGs, *** P < 0.001; (C, D) IHC staining and quantitative analysis showed that MYDGF could decrease the expression of IL-6 of submandibular glands, *** P < 0.001. Scale bars: 100 and 50 μm. Student’s t-test was performed to compare difference between two groups with normal distribution. [file Image2.tif]

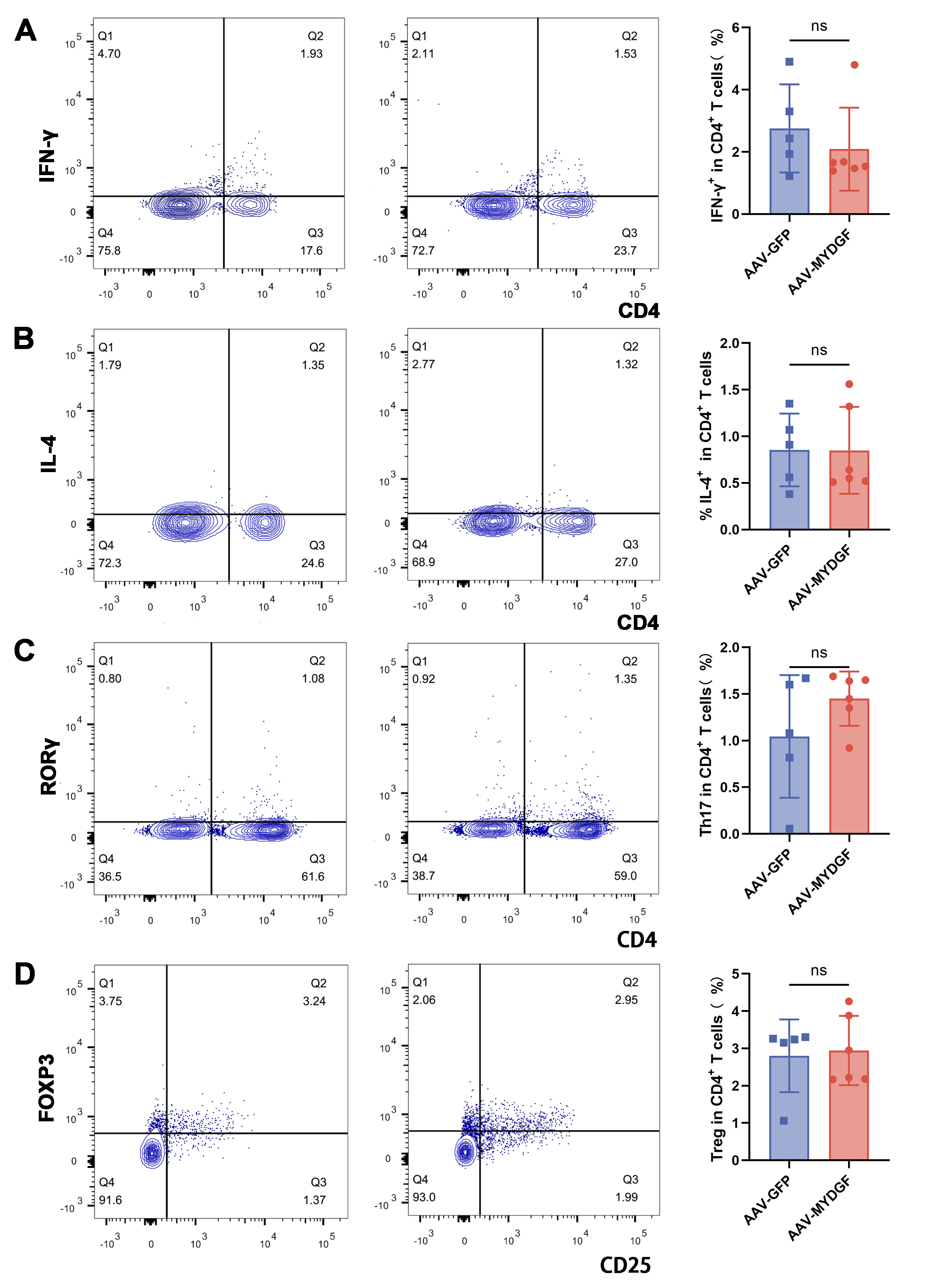

Supplement: Supplementary Figure 3 — The ratio of T helper lymphocyte subsets did not differ in the AAV-MYDGF and AAV-GFP groups. Flow cytometry showed the ratio of (A) Th1, (B) Th2, (C) Th17, (D) Treg were no significantly difference in the AAV-MYDGF and AAV-GFP group, ns no significant difference. Student’s t-test was performed to compare difference between two groups with normal distribution. [file Image3.tif]

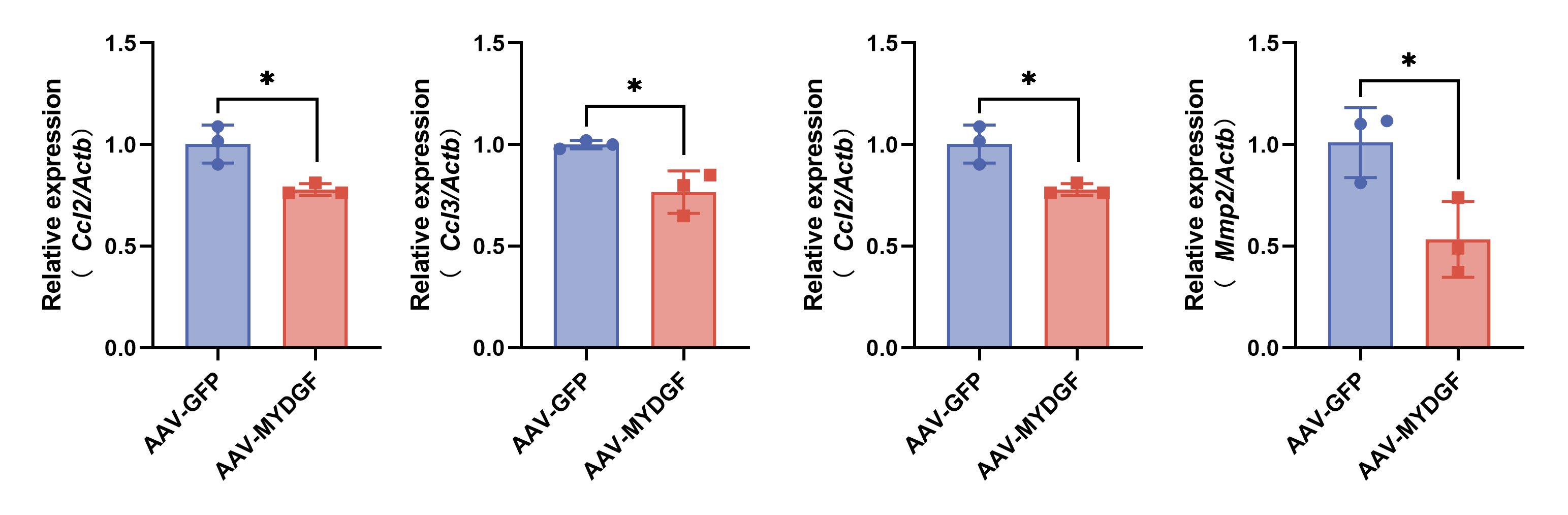

Supplement: Supplementary Figure 4 — Chemokine- and cytokine-related gene of AAV-GFP and AAV-MYDGF group by qRT-PCR. [file Image4.tif]
