# Supplementary material for: Myeloid-derived growth factor promotes M2 macrophage polarization and attenuates Sjögren’s syndrome via suppression of the CX3CL1/CX3CR1 axis
Source: Front Immunol. 2024 Oct 21;15:1465938. doi: 10.3389/fimmu.2024.1465938 (PMC11532040; doi:10.3389/fimmu.2024.1465938)
Supplement: Supplementary file 5 [file Table1.docx]

Table. S1 The primers for specific genes used in qRT-PCR

| Target Gene | Primer sense (5’ - 3’) | Primer antisense (5’ - 3’) |
| --- | --- | --- |
| *Actb* | GGCTGTATTCCCCTCCATCG | CCAGTTGGTAACAATGCCATGT |
| *Cx3cl1* | ACGAAATGCGAAATCATGTGC | CTGTGTCGTCTCCAGGACAA |
| *Cx3cr1* | GAGTATGACGATTCTGCTGAGG | CAGACCGAACGTGAAGACGAG |
| *Ccl2* | TTAAAAACCTGGATCGGAACCAA | GCATTAGCTTCAGATTTACGGGT |
| *Ccl12* | ATTTCCACACTTCTATGCCTCCT | ATCCAGTATGGTCCTGAAGATCA |
| *Ccl3* | TTCTCTGTACCATGACACTCTGC | CGTGGAATCTTCCGGCTGTAG |
| *Mmp2* | ACCTGAACACTTTCTATGGCTG | CTTCCGCATGGTCTCGATG |
